# AON-Mediated Exon Skipping to Bypass Protein Truncation in Retinal Dystrophies Due to the Recurrent *CEP290* c.4723A > T Mutation. Fact or Fiction?

**DOI:** 10.3390/genes10050368

**Published:** 2019-05-14

**Authors:** Iris Barny, Isabelle Perrault, Christel Michel, Nicolas Goudin, Sabine Defoort-Dhellemmes, Imad Ghazi, Josseline Kaplan, Jean-Michel Rozet, Xavier Gerard

**Affiliations:** 1 Laboratory of Genetics in Ophthalmology (LGO), INSERM UMR1163, Institute of Genetics Diseases, Imagine and Paris Descartes University, 75015 Paris, France; iris.barny@institutimagine.org (I.B.); isabelle.perrault@inserm.fr (I.P.); christel.michel@agroparistech.fr (C.M.); josseline.kaplan@inserm.fr (J.K.); 2 Cell Imaging Core Facility of the Structure Fédérative de Recherche Necker, INSERM US24/CNRS UMS3633, Imagine and Paris Descartes University, 75015 Paris, France; nicolas.goudin@inserm.fr; 3 Service D’exploration de la Vision et Neuro-Ophtalmologie, Pôle D’imagerie et Explorations Fonctionnelles, CHRU de Lille, 59037 Lille, France; sabine.defoort@chru-lille.fr; 4 Department of Ophthalmology, IHU Necker-Enfants Malades, 75015 Paris, France; imad.ghazi@aphp.fr; 5 Unit of Retinal Degeneration and Regeneration, Department of Ophthalmology, University of Lausanne, Hôpital Ophtalmique Jules Gonin, Fondation Asile des Aveugles, 1004 Lausanne, Switzerland

**Keywords:** Leber congenital amaurosis and allied retinal ciliopathies, *CEP290*, Flanders founder c.4723A > T nonsense mutation, Cilia elongation, spontaneous nonsense correction, AON-mediated exon skipping

## Abstract

Mutations in *CEP290* encoding a centrosomal protein important to cilia formation cause a spectrum of diseases, from isolated retinal dystrophies to multivisceral and sometimes embryo–lethal ciliopathies. In recent years, endogenous and/or selective non-canonical exon skipping of mutant exons have been documented in attenuated retinal disease cases. This observation led us to consider targeted exon skipping to bypass protein truncation resulting from a recurrent mutation in exon 36 (c.4723A > T, p.Lys1575*) causing isolated retinal ciliopathy. Here, we report two unrelated individuals (P1 and P2), carrying the mutation in homozygosity but affected with early-onset severe retinal dystrophy and congenital blindness, respectively. Studying skin-derived fibroblasts, we observed basal skipping and nonsense associated–altered splicing of exon 36, producing low (P1) and very low (P2) levels of CEP290 products. Consistent with a more severe disease, fibroblasts from P2 exhibited reduced ciliation compared to P1 cells displaying normally abundant cilia; both lines presented however significantly elongated cilia, suggesting altered axonemal trafficking. Antisense oligonucleotides (AONs)-mediated skipping of exon 36 increased the abundance of the premature termination codon (PTC)-free mRNA and protein, reduced axonemal length and improved cilia formation in P2 but not in P1 expressing higher levels of skipped mRNA, questioning AON-mediated exon skipping to treat patients carrying the recurrent c.4723A > T mutation.

## 1. Introduction

Leber congenital amaurosis (LCA, MIM204000) is a group of neonatal-onset and severe retinal dystrophies and a leading cause of incurable blindness in childhood (Frequency 1:30,000; 20% of children attending schools for the blind in western Europe) [1]. It typically occurs as a non-syndromic disease that displays large genetic, allelic and physio–pathological heterogeneity, challenging therapeutic developments [2]. Mutations in *CEP290* (MIM610142) encoding a widely expressed centrosomal protein involved in cilia formation and maintenance [3], are the leading cause of the disease, referred to as LCA type 10 (LCA10; MIM611755) [4,5]. Despite early-onset visual loss, LCA10 individuals display prolonged (>30 years) sparing of central photoreceptors with intact visual brain pathway, creating the conditions to develop therapies built on correcting genetic lesions [6]. A large number of LCA10-causing mutations are reported, including the highly prevalent c.2991 + 1655A > G (p.Cys998*) and c.4723A > T (p.1575Lys*) variants involved in 10% and 2.5% of all LCA cases, respectively [5,7]. The c.2991 + 1655A > G change activates a deep intronic cryptic splice site and introduces a frameshifting pseudo-exon in the mRNA [8,9,10,11]. Antisense oligonucleotides (AONs) have proven effective to redirect the splicing machinery towards the consensus splice sites and bypass protein truncation in primary fibroblasts, IPSC-derived 3D retinal organoids and humanized mice carrying the mutation [8,10,12]. Subsequently, a phase I/II clinical trial (NCT03140969) has been launched which demonstrated safety and clinical relevance (vision improvement) of intravitreal injections of splice-modulating oligonucleotide [13]. The c.4723A > T variant, like the vast majority of *CEP290* mutations, is predicted to truncate the protein and is amenable to gene augmentation therapy. However, this approach is challenging due to both the *CEP290* cDNA size (7.4 Kb) which over-exceed cargo capacities of AAV vectors (<5 Kb) preferred in the field of retinal diseases [14,15,16] and risk of overexpression toxicity [17,18]. Interestingly, consistent with an important role in cilia metabolism, *CEP290* mutations have been associated with additional human phenotypes, including oculo-renal Senior Loken syndrome (SLSN6, MIM610189), oculo-cerebro-renal Joubert syndrome (JBTS5, MIM610188) and embryo-lethal Meckel syndrome type 4 (MKS; MIM611134) [19]. Observation of endogenous basal exon-skipping producing low-levels of alternatively spliced coding *CEP290* mRNAs has inspired a model of pathogenesis according to which disease severity is a function of the amount of CEP290 a cell can produce from mutant alleles [20]. Consistently, low levels of premature termination codon (PTC)-free *CEP290* mRNA produced by endogenous basal alternative splicing and/or nonsense associated altered splicing have been identified in fibroblasts from individuals with biallelic *CEP290* truncating mutations but mild retinal phenotypes [21,22,23]. Lessening the disease through somatic frame-restoration mechanisms is reminiscent of genetic reversion in dystrophin-positive muscular fibers from individuals with Duchene muscular dystrophy which inspired AON-mediated exon skipping to bypass dystrophin truncation and switch the disease to attenuated Becker muscular dystrophy. Following this example, we considered AON-mediated skipping of *CEP290* exon 36 to bypass protein truncation resulting from the c.4723A > T nonsense mutation. Here, studying fibroblasts from two unrelated individuals homozygous for the mutation and controls, we show combination of endogenous and selective exon skipping producing a minimally shortened *CEP290* mRNA and a protein that localized at the centrosome. Cilia analysis revealed no major anomaly but a significant axonemal elongation. Using AON specific to the donor consensus splice-site of exon 36 in patient and control fibroblasts, we were able to increase the abundance of the alternatively spliced mRNA and shortened protein and to reduce axonemal length. However, fibroblasts with highest levels of alternatively spliced products displayed reduced ability to ciliate, questioning the relevance of AON-mediated exon skipping to treat patients carrying the recurrent c.4723A > T mutation.

## 2. Materials and Methods

### 2.1. Genetic Analysis

P1 and P2, two unrelated simplex cases born to apparently non-consanguineous parents originating from the transnational Flanders region were addressed to the Molecular Diagnosis Unit of our Genetic Department for molecular diagnosis of early-onset and severe retinal dystrophy. Patient DNAs were subjected to panel-based molecular testing of 199 genes involved in retinal dystrophies (Figure S1) and variant datasets were filtered using the Polydiag software of the Polyweb series developed in-house. Biallelism for apparently homozygous *CEP290* c.4723A > T variant was assessed by Sanger sequencing of parental DNA using primers specific to *CEP290* exon 36 (Supplementary Materials, Table S1). Written informed consents were obtained from all participating individuals and the study was approved by the Comité de Protection des Personnes “Ile-De-France II” (3 March 2015/DC 2014–2272).

### 2.2. In Silico Analysis of the Nonsense c.4723A > T (p.Lys1575*) Mutation on Splicing

The consequence of the c.4723A > T substitution on splicing was assessed using several prediction softwares, as previously described [21].

### 2.3. Cell Culture

Fibroblast cell lines were derived from skin biopsies of affected subjects (P1 and P2) and three healthy individuals (C1–C3). A table recapitulating their genetic and clinical features is presented in Table S2. Primary fibroblasts (<15 passages) were cultured as previously described [21].

### 2.4. AON and Transfection

AON specific to the donor splice site of *CEP290* exon 36 was identified using software prediction tools (m-fold and ESEfinder3.0 programs available online at http://mfold.rna.albany.edu/ and http://rulai.cshl.edu/cgi-bin/tools/ESE3/esefinder.cgi respectively) and following general recommendations [24].

The sequence of H36D (+98 −11) AON was as following: 5′-UAGAAUCUUACCCAAGCCGUUU-3′.

This AON was synthetized by Eurofins Genomics (St. Quentin Fallavier, France) and contains 2′-O-methyl RNA and full-length phosphorothioate backbone. Cells at 80% confluence were transfected with different concentrations of H36D AON, ranging from 20–300 nmol/L, in Opti-MEM supplemented with 10% fetal bovine serum using Lipofectamine2000 (Invitrogen, Carlsbad, CA, USA) according to the manufacturer’s instruction. Cells were harvested for mRNA or protein analysis between 4 and 72 h.

### 2.5. RNA Analysis

RNA isolation from fibroblasts and wild-type human fetal retina (22 weeks) and cDNA synthesis were performed according to previously described methods [21].

Reverse transcription-PCR (RT-PCR) was performed using 2 µL of cDNA as described previously [21]. *CEP290* splicing isoforms were amplified using primer pairs specific to exons 35 and 37 (Figure S2 and Table S3) and PCR products were resolved in a 3% agarose gel.

For real-time quantitative PCR (RT-qPCR), cDNA (5 µL of a 1:25 dilution) were amplified using primers specific to the full-length and skipped isoforms, respectively (Figure S2 and Table S4), according to the protocol described in Barny et al. 2018. The human *β*-glucuronidase (*GUSB*, NM_000181.3) and P0 large ribosomal protein (*RPLP0*, NM_001002.3) mRNAs were used to normalize the data. The data were analyzed as described previously [8].

### 2.6. Protein Analysis

For Western blot analysis, total proteins from treated and untreated cells were extracted and quantified as described previously [21], and the relative abundance of CEP290 protein was estimated by densitometry using *β*-Actin as reference in each cell line.

For immunocytochemistry analysis, cells were grown for 24 h on coverslips in 12-well plates to reach 90–100% confluence and either transfected using the H36D (+98 −11) AON or left untreated. After 24 h, treated and untreated cells were serum-starved for 48 to 72 h before cold methanol fixation and immune-labeling of ARL13B, CEP290, CP110, IFT88, pericentrin, RAB8A, *γ*-Tubulin and/or acetylated *α*-Tubulin. Immunofluorescence images were acquired and processed to analyze cilia abundance, axonemal length, subcellular localization and/or staining intensities. All experimental procedures and analytical methods were described in Barny et al., 2018.

### 2.7. Statistics

All statistical analyses were run using the Prism6 software, and the significance was determined using one-way ANOVA with post hoc Tukey’s test. The data obtained from C1–C3 were systematically pooled for immune-labelling analysis. Error bars reflect the standard errors for the mean (SEMs).

## 3. Results

### 3.1. Panel-Based Molecular Diagnosis Testing Identifies Homozygosity for the CEP290 c.4723A > T Founder Mutation in Two Individuals with Congenital Retinal Dystrophy of Variable Severity

We studied two apparently unrelated non-consanguineous sporadic cases of Belgian and/or French Flanders origin addressed for congenital retinal dystrophy with no extraocular involvement. The first individual (P1) presented at birth with nystagmus, photoaversion, hyperopia (+6 diopters LRE), absent cone-derived electroretinogram but present, yet highly hypovolted, rod-derived responses. He experienced spontaneous improvement of his visual capacities in the first decade of life. At the age of 20 years, he displayed a tubular visual field with a visual acuity (VA) of 20/67 (RE) and 20/50 (LE) and thin retinal vessels, optic nerve atrophy and peripheral pigmentary deposits at the fundus. While the initial symptoms suggested early-onset severe cone-rod dystrophy, this outcome is consistent with a rod-dominant LCA-like disease referred to as early-onset severe rod-cone dystrophy. The second individual (P2) presented with a typical LCA10-associated disease, i.e., a stationary congenital blindness with nystagmus, inability to follow lights or objects and flat cone- and rod-derived electroretinographic responses. Panel-based molecular diagnosis for inherited retinal dystrophies (199 genes) and Sanger-based familial segregation analysis identified homozygosity for the Flanders founder *CEP290* c.4723A > T (p.Lys1575*) mutation in the two cases.

### 3.2. In Silico Analysis Suggests That the CEP290 c.4723A > T Mutation Induced Nonsense-Associated Altered Splicing

We analyzed the effect of the c.4723A > T mutation on splicing by using prediction software solutions scrutinizing splice signals and exonic splicing silencer (ESS)/exonic splicing enhancer (ESE) binding sites. The mutation had no effect on the canonical splice sites of exon 36 (not shown), but it increased the exon 36 ESS/ESE ratio, thus elevating the susceptibility of skipping compared to the wild-type sequence (Table 1). The substitution of the c.4723 adenine into a guanine but not a cytosine is predicted to have the same effect.

### 3.3. mRNA Analysis Supports c.4723A > T-Mediated Nonsense-Associated Altered Splicing and Basal Endogenous Alternative Splicing of Exon 36

**Figure 1:**
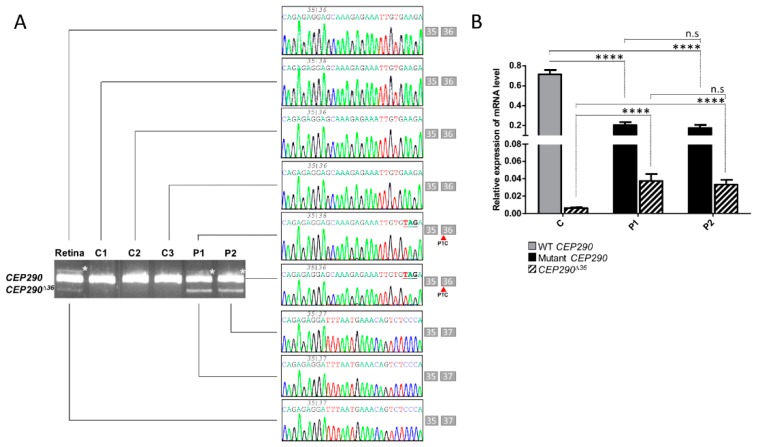
Naturally occurring exclusion of *CEP290* exon 36 encompassing premature stop codon. (**A**) Analysis of *CEP290* transcript extracted from wild-type human fetal retina (Retina), control (C1–C3) and patient (P1 and P2) cell lines. Image of agarose gel and Sanger sequencing electropherograms showing amplicons produced using primer pairs surrounding mutant exon 36 and corresponding sequences. The boxes close to electropherograms summarize the exonic organization and phasing of each reverse transcription (RT)-PCR fragment. White asterisks point to heteroduplex products. Red arrows show the position of the premature termination codon (PTC) within exon 36 *CEP290* isoform. (**B**) Relative expression levels of WT (grey bar) and mutant (black bars) full-length and skipped (*CEP290^∆36^*; hatched bars) *CEP290* mRNAs in control (C represents pooled values of C1–C3) and patient (P1 and P2) fibroblasts as determined by RT-qPCR. Bars correspond to the mean ± SEM derived from ten independent experiments. **** *p* ≤ 0.0001, n.s = not significant.

Agarose gel analysis and Sanger sequencing of RT-PCR products generated from P1 and P2 skin fibroblast mRNAs carrying the c.4723A > T variant in homozygosity using primers specific to *CEP290* exons 35 and 37 (Figure S2 and Table S3) detected the full-length mutant cDNA (*CEP290*) and a PTC-free alternatively spliced product lacking exon 36 (*CEP290^∆36^*). Control fibroblasts expressed the full-length wild-type cDNA but the *CEP290^∆36^* product was undetectable, contrasting with human retina where both isoforms were identified. These observations indicate that *CEP290* exon 36 undergoes endogenous basal skipping in the retina and that, consistent with in silico analysis, the c.4723A > T variant induces nonsense-associated altered splicing (Figure 1A). Interestingly, RT-qPCR analysis using primers specific to the *CEP290^∆36^* isoform (Figure S2 and Table S4) detected the product in control fibroblasts (Figure 1C). Consistent with expression below the threshold of agarose gel detection, the abundance of the *CEP290^∆36^* mRNA in controls was approximately one-tenth that measured in patient cells (Figure 1C). This observation supports some contribution of endogenous basal exon skipping in *CEP290*-frame-restoration documented in P1 and P2 mutant fibroblasts. RT-qPCR analysis using primers specific to the full-length mutant/wild-type cDNA (Figure S2 and Table S4) showed reduced abundance of the mutant product in P1 and P2 cell lines compared to the wild-type counterpart in controls supporting nonsense-mediated RNA decay (NMD) of the mRNA carrying the nonsense c.4723A > T variant (Figure 1B).

### 3.4. Protein Analysis Detects Low Levels of a CEP290 Protein That Localizes at the Centrosome in Patient Fibroblasts Homozygous for the c.4723A > T Nonsense Mutation

**Figure 2:**
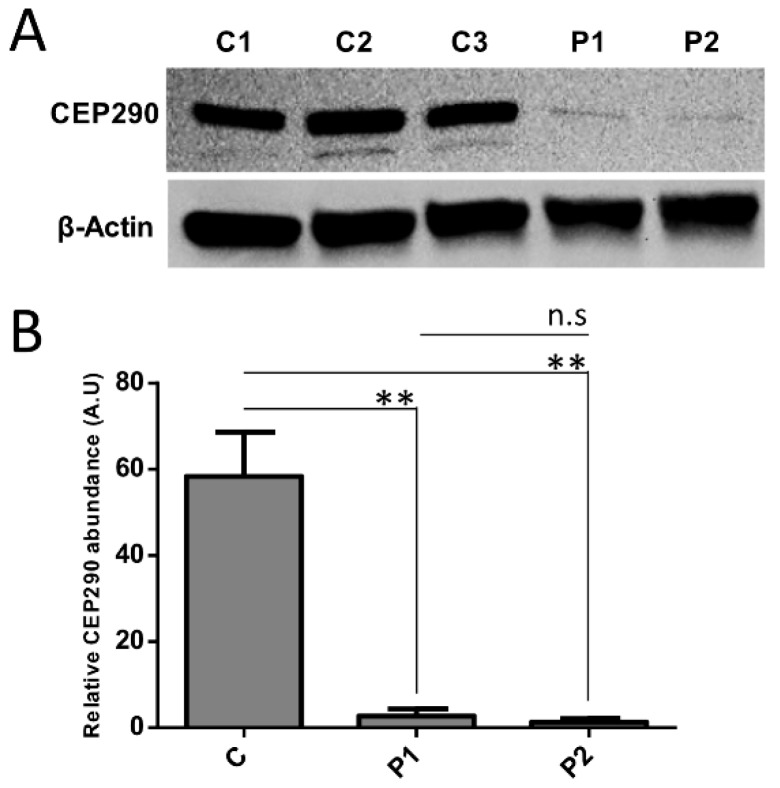
Effect of the natural exclusion of PTC-encoding exon 36 on CEP290 protein production. (**A**) Immune detection of the CEP290 and *β*-Actin in control cell lines (C1–C3) and mutant fibroblasts (P1 and P2). (**B**) Quantification of CEP290 abundance relative to *β*-Actin in control (C represents the pooled values of C1–C3) and patient fibroblasts. Bars correspond to the mean value ± SEM from four independent experiments. ** *p* ≤ 0.01, n.s = not significant.

**Figure 3:**
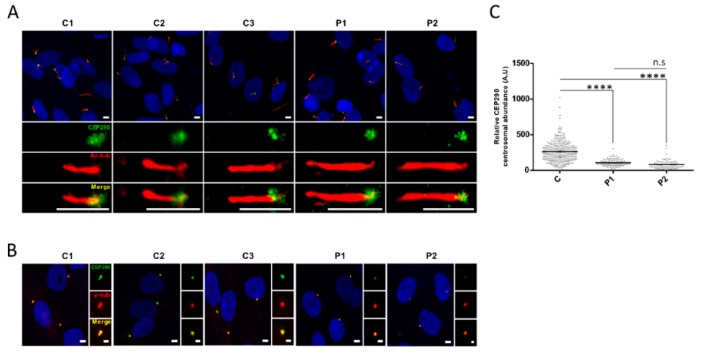
CEP290 expression assessment in quiescent cells. (**A**) Representative images of CEP290 (green) localization in quiescent control and mutant fibroblasts. Acetylated *α*-tubulin (Ac-tub; red) is used to stain the ciliary axoneme. As in control cell lines (C1–C3), CEP290 is correctly localized at the base of the cilia in patient (P1 and P2) fibroblasts. Scale bar, 5 µm. (**B**) Centrosomal localization of CEP290 (green) in control and mutant fibroblasts. The *γ*-tubulin (*γ*-tub; red) labeling is used as a centrosomal marker. Image scale bar, 5 µm and inset scale bar, 2 µm. (**C**) Quantification of the CEP290 immunofluorescence intensity at the basal body in each cell line (C represents the pooled values of C1–C3). Each dot depicts the labeling intensity of the protein in individual cells from six microscope fields (recorded automatically). The solid line indicates the mean. **** *p* ≤ 0.0001, n.s = not significant. A.U. = arbitrary unit.

Western blot analysis of protein extracts from serum-starved P1 and P2 fibroblasts detected minimal amounts of a CEP290 product around 290 KDa (Figure 2 and Figure 3C) that localized at the centrosome upon immunocytochemistry analysis (Figure 3A,B). These results indicate that the PTC-free alternatively spliced product lacking exon 36 was translated into a stable protein that can be recruited at the centrosome as does the wild-type protein.

### 3.5. Cilia Analysis of Serum-Starved Patient Fibroblasts Shows Apparently Normal RAB8A Localization at the Centrosome but Elongated Axonemes

**Figure 4:**
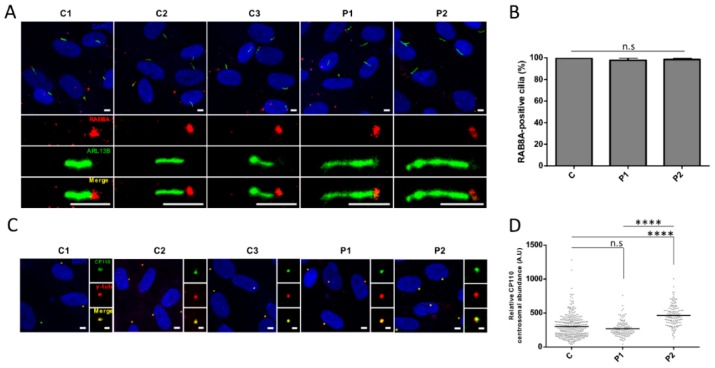
Localization and abundance of CEP290 centriolar satellite partners. (**A**) Representative images of RAB8A (red) localization in the cilia from control (C1–C3) and mutant (P1 and P2) fibroblasts induced to quiescence. ARL13B (green) labeling is used to mark the ciliary membrane. Scale bar, 5 µm. (**B**) Quantification of RAB8A-positive cilia in each cell line (C represents the pooled values of C1–C3). Bars correspond to the mean ± SEM (*n* ≥ 50 cilia for each group). n.s = not significant. A.U. = arbitrary unit. (**C**) Representative images of CP110 (green) centrosomal staining in quiescent control and mutant fibroblasts. Centrosomes are stained by γ-tubulin (*γ*-tub.; red). Image scale bar, 5 µm and inset scale bar, 2 µm. (**D**) Quantification of CP110 immunofluorescence intensity at centrosomes in quiescent fibroblasts (C represents the pooled values of C1–C3). Each dot depicts the labeling intensity of the protein in individual cells from six microscope fields (recorded automatically). The solid line indicates the mean. **** *p* ≤ 0.0001, n.s = not significant. A.U. = arbitrary unit.

**Figure 5:**
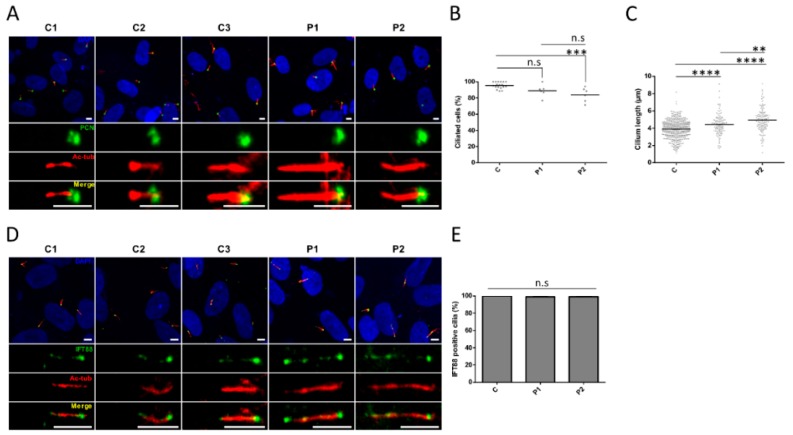
Ciliogenesis and axonemal trafficking. (**A**) Representative images of cilia in the quiescent control (C1–C3) and mutant (P1 and P2) fibroblasts. The cilium axoneme is labeled with acetylated *α*-tubulin (Ac-tub; red) and the basal body using pericentrin as a marker (PCN; green). Scale bar, 5 µm. (**B**) Percentage of ciliated cells and (**C**) length of cilia axonemes in control and patient fibroblasts. A minimum of 90 ciliated cells were considered for each cell lines. (**D**) Representative images of IFT88 (green) localization along the cilium in quiescent control (C1–C3) and mutant (P1 and P2) fibroblasts. Acetylated *α*-tubulin (Ac-tub; red) staining was used as a marker of the ciliary axoneme. Scale bar, 5 µm. (**E**) Quantification of IFT88-positive cilia. Bars represent the mean ± SEM (*n* ≥ 80 cilia for each group). C regroups the values of C1–C3. ** *p* ≤ 0.01, *** *p* ≤ 0.001, **** *p* ≤ 0.0001, n.s = not significant.

*CEP290* exon 36 encodes 36 amino-acids contributing to the CEP290 domain that binds RAB8A, the recruitment of which at the centrosome leads to the release of the cilia formation-suppressor CP110, hence initiating ciliogenesis during transition of the cells from proliferation to quiescence [25,26,27]. Interestingly, RAB8A immune-labeling in quiescent fibroblasts showed comparable localization at the basal body in patient and control fibroblasts, suggesting that the absence of information encoded by exon 36 does not alter the recruitment of RAB8A at the centrosome (Figure 4A,B). Furthermore, we observed comparable CP110 abundance at the centrosome of control and P1 fibroblasts indicating correct release upon RAB8A recruitment (Figure 4C,D). In contrast, the amount of CP110 at the centrosome of P2 cells was significantly higher than in control and P1 cells (*p* ≤ 0.0001; Figure 4C,D). Whether CP110 accumulation in P2 could be correlated to reduced abundance of CEP290^∆36aa^ protein compared to P1 is possible but remains hypothetic as the difference in CEP290^∆36aa^ amounts was not statistically significant between patient cell lines. Consistent with normal and impaired CP110 release, cilia abundance was in the normal range and reduced in P1 and P2 fibroblasts, respectively (*p* ≤ 0.001; Figure 5A,B).

On another note, measuring cilia length, we observed statistically significant axonemal elongation in both patient cell lines compared to controls (mean axonemal sizes of 4.4 µm in P1 and 4.9 µm in P2 versus 3.9 µm in controls, *p* ≤ 0.0001; Figure 5A,C). Cilia in P2 expressing lower amounts of the CEP290^∆36aa^ isoform at the centrosome displayed significantly longer axoneme than P1 counterparts (*p* ≤ 0.01; Figure 5A,C), further supporting a possible correlation between the severity of cilia phenotype and the amount of minimally shortened CEP290 cells are able to produce from mutant alleles.

As observed in controls cells, IFT88 immune-labelling in patient fibroblasts revealed this intraflagellar trafficking (IFT) complex B protein all along the axoneme (Figure 5D,E), assuming that the abnormal cilia elongation in patient cells is not related to a defect of the anterograde trafficking driven by IFT88.

### 3.6. Targeting the Consensus Donor Splice Site Enables Dose- and Time-Dependent Skipping of CEP290 Exon 36

**Figure 6:**
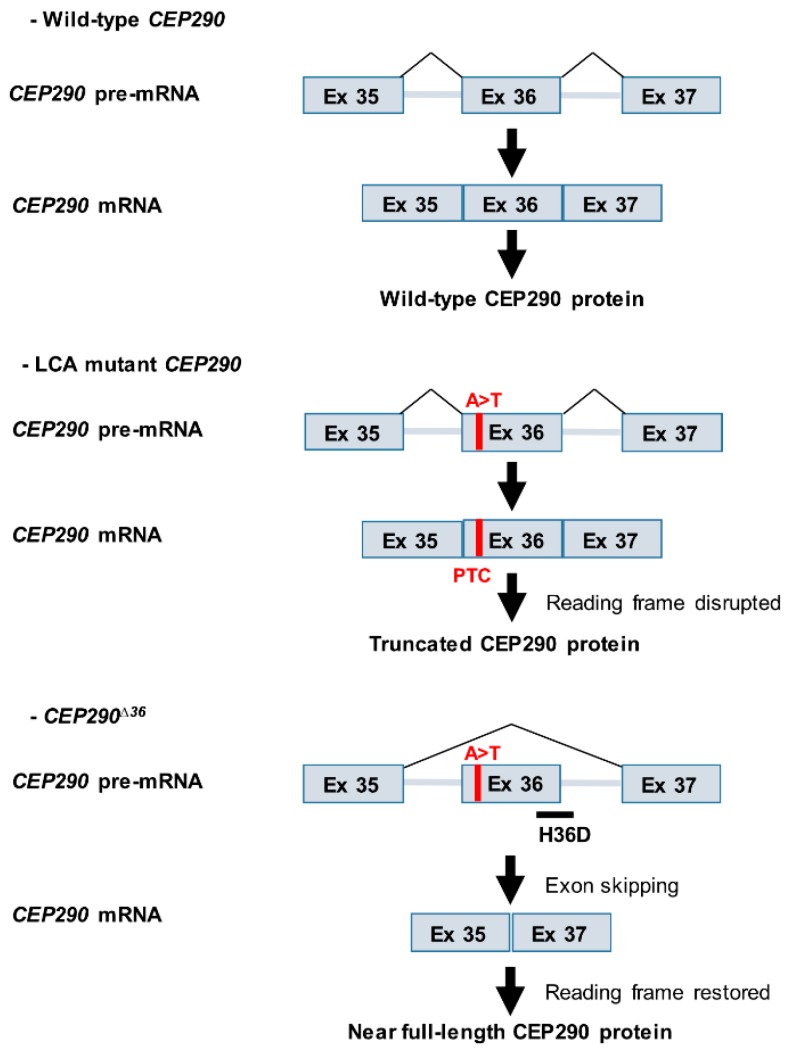
Antisense oligonucleotides (AONs)-mediated splicing alteration of *CEP290* pre-mRNA to bypass protein truncation. An exonic mutation in *CEP290* pre-mRNA (c.4723A > T, in red) introduces a PTC within exon 36 to *CEP290* mRNA. Administration of AON (in black), targeting the splice donor site (H36D), is predicted to alter splicing by blocking the recognition of exon 36. Exclusion of exon 36 (*CEP290^∆36^*) should allow bypassing protein truncation while maintaining the reading frame, and lead to the production of near full-length CEP290 protein.

**Figure 7:**
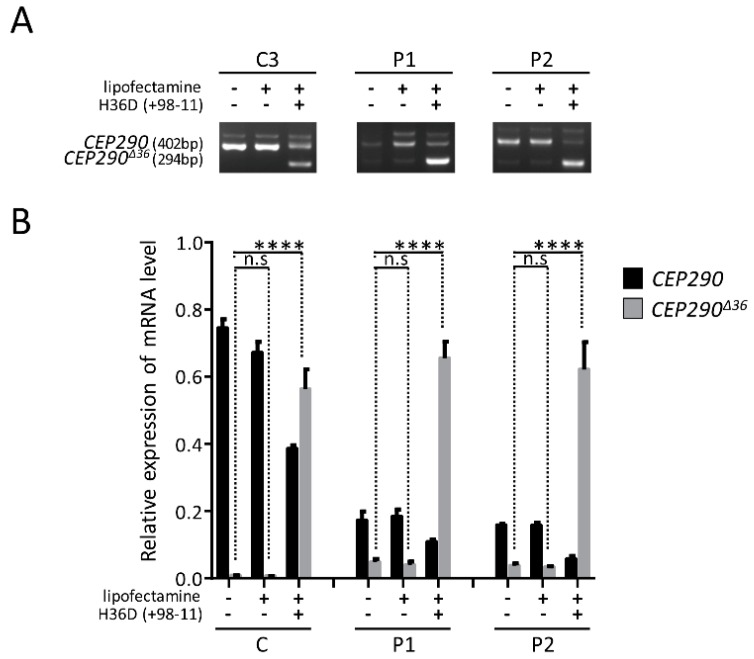
Effect of AON-mediated exon 36 skipping on *CEP290* mRNAs. (**A**) RT-PCR analysis of *CEP290* transcripts expressed in control (C3) and patient (P1 and P2) fibroblasts untreated or treated for 24h with lipofectamine alone or associated to H36D AON. Images of agarose gel showing amplicons produced using primer pairs surrounding mutant exon 36. (**B**) Relative expression levels of full-length (black bars) and exon 36-skipped (grey bars) *CEP290* mRNAs in control (C1–C3) and patient (P1 and P2) fibroblasts as determined by RT-qPCR. Bars show the mean ± SEM from three independent experiments. C represents the pooled values of C1–C3. **** *p* ≤ 0.0001, n.s., not significant.

The RNA conformation around *CEP290* exon 36 and splicing regulatory elements were predicted in silico using m-fold and ESEfinder3.0 programs to design splice switching AON. We designed 2′-O-methyl-phosphorothioate (2′-OMePs) AON targeting the donor site (H36D (+98-11)) (Figure 6). The AON was delivered in patient and control fibroblasts at a final concentration of 150nM for 24h prior to mRNA analysis. Treatment with H36D (+98 − 11) AON elevated significantly the abundance of products lacking exon 36 and reduced by half the amount of full-length mutant and wild-type products in patient and control fibroblasts, respectively (Figure 7). Due to NMD, the abundance of the full-length mutant was significantly reduced in patient compared to control cells (Figure 7B). Consistent with a switch from a mRNA prone to NMD to a PTC-free isoform, the abundance of the alternatively spliced product lacking exon 36 in patient fibroblasts was comparable to that of controls upon treatment with the H36D (+98 − 11) AON. Treatment with the transfection reagent alone did not alter *CEP290* expression whatever the cell line (Figure 7).

**Figure 8:**
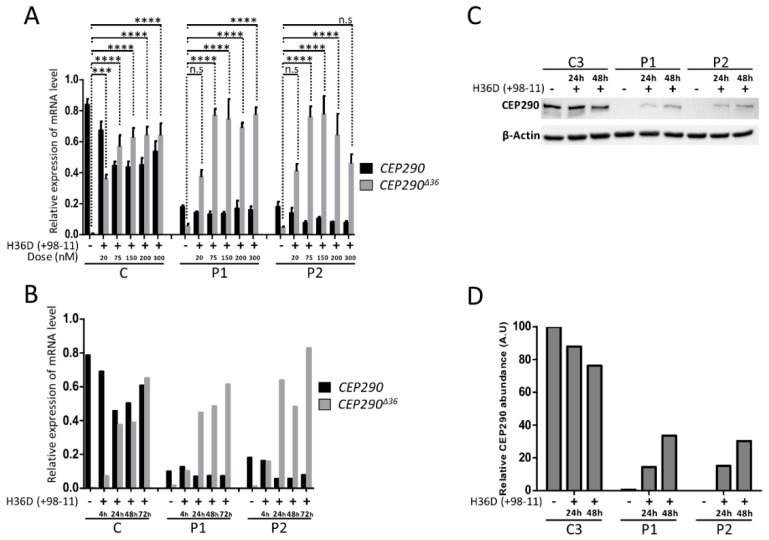
Optimization of transfection conditions. (**A**) RT-qPCR analysis of reverse transcribed *CEP290* mRNA extracted from control (C1–C3) and patient (P1 and P2) fibroblasts untreated or after transfection of increasing doses (20 nM to 300 nM) of H36D oligonucleotide. The graph shows the mean amounts (±SEM) of full-length (*CEP290*; black bars) and exon 36-skipped transcripts (*CEP290^∆36^*; grey bars) from three independent experiments. C regroups the values obtained for C1–C3. *** *p* ≤ 0.001, **** *p* ≤ 0.0001, n.s., not significant. (**B**) RT-qPCR analysis of reverse transcribed *CEP290* mRNA extracted from control (C1–C3) and patient (P1 and P2) fibroblasts untreated or treated during increasing times of treatment (4 h to 72 h) with 75nM of H36D oligonucleotide. The graph shows the amounts of full-length (*CEP290*; black bars) and exon 36-skipped transcripts (*CEP290^∆36^*; grey bars). C corresponds to C1–C3 pooled values. (**C**) CEP290 protein analysis in control (C3) and mutant (P1 and P2) cell lines untreated or treated with 75nM of H36D during 24 h or 48 h. (**D**) Relative quantification of CEP290 protein abundance depending on treatment time. *β*-Actin was used for normalization.

To assess dose-dependent skipping efficiency, patient and control fibroblasts were treated with increasing doses of H36D (+98 − 11) AON for 24 h, revealing that the amount of alternatively splice products lacking exon 36 reached a maximum in almost all cell lines at an AON concentration of 75 nmol/l (Figure 8A). At this concentration, we observed accumulation of alternatively splice products and CEP290 proteins with treatment time (Figure 8B–D).

### 3.7. AON-Mediated Skipping Allows to Bypass Protein-Truncation but May Not Restore Full CEP290 Functions

**Figure 9:**
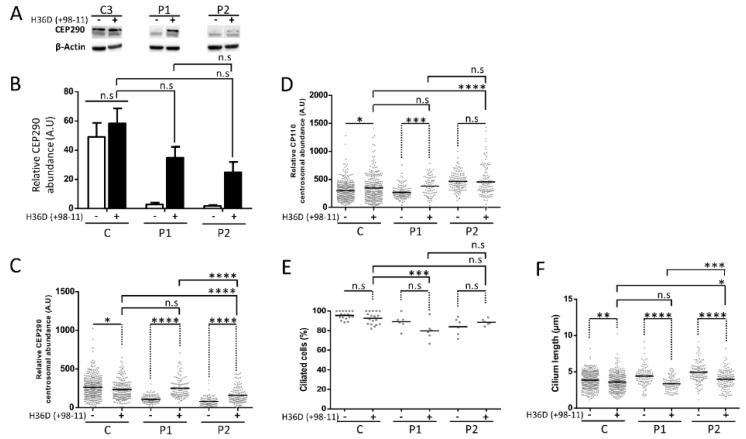
AON-treatment effect at protein level and impact on ciliation. (**A**) CEP290 protein analysis in control and mutant cell lines untreated or treated during 48 h with 75 nM of H36D. (**B**) Quantification of CEP290 protein abundance relative to *β*-Actin. Bars correspond to the mean value ± SEM from three independent experiments = not significant. A.U. = arbitrary unit. (**C**) Quantification of CEP290 immunofluorescence intensity at the basal body in each cell line. Each dot depicts the labeling intensity of the protein in individual cells recorded automatically from six microscope fields. The solid line indicates the mean. (**D**) Quantification of CP110 immunofluorescence intensity at centrosomes in quiescent fibroblasts. All automatic intensity measures were recorded from six fields. (**E**) Percentage of ciliated cells and (**F**) length of cilia axonemes in control and mutant fibroblasts. A minimum of 90 ciliated cells were considered for each cell lines. C corresponds to C1–C3 pooled values. * *p* ≤ 0.05, ** *p* ≤ 0.01, *** *p* ≤ 0.001, **** *p* ≤ 0.0001, n.s = not significant, A.U. = arbitrary unit.

The abundance of full length *CEP290* mRNA in control cells treated with 75nM of H36D (+98 − 11) AON for 48 h was around 60% that of untreated control cells, as determined by RT-qPCR (Figure 8B), indicating that approximately 40% of full length *CEP290* pre-mRNA underwent AON-mediated skipping of exon 36. The amount of CEP290 protein (full-length + ∆36aa) was comparable in treated and untreated cells (Figure 9A,B, left), suggesting that the CEP290^∆36aa^ isoform is stable. Yet, treated cells displayed a moderate diminution of CEP290 staining at the centrosome (*p* ≤ 0.05, Figure 9C) with statistically significant, yet minimal, alteration of CP110 centrosomal staining (*p* ≤ 0.05, Figure 9D). The abundance of ciliated cells tended to be slightly diminished (95.5% versus 92.5%, Figure 9F) as was the mean axonemal length (3.9 µm versus 3.6 µm, *p* ≤ 0.01, Figure 9E). Together, these results suggest that the CEP290^∆36aa^ isoform interferes with the wild-type counterpart and can compromise ciliation, possibly through disorganization of centriolar satellites. The same treatment in P1 cells, allowed a highly significant increase in CEP290^∆36aa^ protein abundance, as determined by western blot and immunocytochemistry analyses (Figure 9A–C). Interestingly, the intensity of CEP290 staining at the centrosome reached that of treated control cells (Figure 9C). However, expressing increased levels of CEP290^∆36aa^ isoform in cells deprived of wild-type CEP290 altered the dynamics of centriolar satellites, as documented by increased dispersion of CP110-specific centrosomal staining (*p* ≤ 0.001, Figure 9D). Consistently, the proportion of ciliated cells tended to diminish (89% versus 79.8%) upon AON treatment (Figure 9F), as did axonemal length (4.4 µm versus 3.5 µm, *p* ≤ 0.0001, Figure 9E). In P2, AON treatment also led to a significant increase in the centrosomal abundance of the CEP290^∆36aa^ isoform (*p* ≤ 0.0001, Figure 9C). However, the amount of this product was significantly reduced compared to the counterpart in treated P1 cells (*p* ≤ 0.001). We observed a reduced impact of the treatment on centriolar satellites (Figure 9D) compared to P1, cilia abundance tended to minimally increase (Figure 9F) and cilia length significantly decreased (*p* ≤ 0.0001, Figure 9E).

## 4. Discussion

Targeted exon skipping is gaining a growing interest in rare hereditary diseases involving cell types as diverse as myocytes, keratinocytes or motor neurons. Recently, renal cells were added to this list with the report on AON-mediated rescue of Joubert phenotypes in patient-derived primary tubular cells and in a murine model with *CEP290* mutations [28]. Photoreceptors of these individuals and more generally of individuals carrying PTCs in skippable *CEP290* exons certainly deserve consideration. Here, we used skin-derived fibroblasts from two unrelated individuals carrying a *CEP290* nonsense founder mutation to implement AON-mediated frame-restoration to non-syndromic retinal diseases. CEP290 is expressed in the transition zone at the base of cilia of multiple cells systems. There, it bridges the cilia membrane and the axoneme by binding the cellular membrane to microtubules through sequences in the amino-terminal and myosin-tail homology domains, respectively [29]. The integrity of this bridge, known as the ciliary gate CEP290 [3], is essential to allow proper ciliation as demonstrated by reduced cilia formation and/or aberrant axonemal ultrastructure in cells from patients carrying *CEP290* mutations [8,19] and in cellular and in-vivo models of *Cep290* knockdown [10,30,31,32]. Studying the ciliation ability of fibroblasts from the two individuals carrying the c.4723A > T mutation, we observed normal to discreetly reduced abundance of ciliated cells upon serum starvation, suggesting the production of a CEP290 protein from mutant alleles. Accordingly, we observed expression of an alternatively spliced *CEP290* mRNA lacking exon 36 encompassing the PTC and low levels of CEP290 protein. The PTC-free mRNA product was detected both in patient and control cells, though with minimal amounts in controls. This observation indicates (*i*) that *CEP290* exon 36, like exons 6, 10, 18, 32, 41, 46, and 51 [20,21,33], undergoes endogenous non-canonical basal exon skipping in wild-type skin fibroblasts and (*ii*) that the A to T transversion at position c.4723 enhances skipping. In silico analysis which predicted that the variation lies within an ESE indicates that skipping augmentation is promoted through nonsense-associated altered splicing. Indeed, ESE sequences are usually purine- or A/C-rich [34,35]. Introducing a T into an artificial polypurine sequence mimicking a *DMD* ESE has been reported to reduce enhancer activity, in particular when it introduced a nonsense codon [36]. Accordingly, the introduction of a T at position c.4723 is predicted to reduce significantly the strength of ESE and increase the chance of exon skipping, whereas the introduction of a G and a C has a minimal and no effect, respectively (Table 1).

During the transition of cells from proliferation to quiescence, ciliation is initiated by CEP290-mediated recruitment of RAB8A at the centrosome, leading to the release of the cilia formation suppressor CP110 [25,26,27]. Preserved ability of P1 and P2 fibroblasts to ciliate upon serum starvation indicated that the *CEP290^∆36^* mRNA was translated into a protein isoform able to recruit RAB8A despite the loss of 36 CEP290 residues involved in binding. Accordingly, immune-staining demonstrated the presence of CEP290 and RAB8A at the centrosome. The abundance of CEP290 in P1 and P2 cells were 40% and 30% of the wild-type levels in controls, respectively. Despite reduced CEP290 abundance, the intensity of CP110-specific staining at the centrosome of P1 cells was comparable to controls, indicating a normal release that was further supported by a normal abundance of ciliated cells. In contrast, P2 cells which tended to express slightly less *CEP290^∆36^* mRNA and CEP290^∆36aa^ protein exhibited moderate, yet statistically significant, reduction in CP110 release and, accordingly, in cilia abundance. As to why P2 cells tend to express diminished amounts of *CEP290^∆36^* isoform, the reduced abundance of full length mutant mRNA in P2 compared to P1 suggests a depletion of full length mutant pre-mRNA from which the skipped isoform arises. Whether this observation would result from increased NMD in P2, similar amounts of *CEP290^∆36^* isoform should be measured in the two cell lines. Further studies using NMD inhibitors could help in addressing this interesting question. Whatever the mechanism, these results further support the predictive model of *CEP290*-associated pathogenesis and it would be tempting to correlate the severity of the retinal disease of P2 compared to P1 (congenital blindness vs. measurable VA of 20/67 (RE) and 20/50 (LE) at 20 years, respectively) to a reduced abundance of CEP290^∆36aa^ product in photoreceptor cells. Evidence of abundant endogenous basal skipping of exon 36 in human retina makes this hypothesis plausible. However, this cannot be demonstrated, in particular since the level of endogenous and selective (if any) skipping of exon 36 in the photoreceptors of affected individuals cannot be anticipated from the *CEP290* genotype as determined by sequencing of exon and intron boundaries only. Variants in other *CEP290* regions and/or in genes encoding proteins important to endogenous and/or selective exon skipping, the mechanisms of which are not or poorly known, could contribute to the individual *CEP290* pre-mRNA splicing variability. Along the same lines, it is worth mentioning that three individuals carrying the c.4723A > T mutation were reported to present with LCA and intellectual disability, autistic behavior or cerebro-renal anomalies consistent with Joubert syndrome [5,7,37]. Whether the extraocular expression in these individuals is due to reduced skipping of exon 36 in the brain and/or the kidney or to additional mutations is opened to debate. Further, consistent with a correlation between the abundance of centrosomal CEP290 and the cilia phenotype of fibroblasts, AON-mediated augmentation of the amount of CEP290^∆36aa^ at the centrosome of P2 fibroblasts improved the ciliary phenotype. However, a more important increase in P1 cells aggravated the cilia phenotype, supporting the view that the *CEP290^∆36^* mRNA encodes an imperfectly functional protein, the expression of which might be preferable compared to knock-out, provided it is not too high. This indicates that exon 36 encodes an important functional region of CEP290. The centrosomal depletion of CEP290 in control cells expressing significant levels of *CEP290^∆36^* mRNA (40% of total CEP290) upon AON treatment, suggests that the CEP290^∆36aa^ has reduced ability to associate to the centrosome, predicting a diminution of RAB8A recruitment, possibly aggravated by the loss of 36 amino-acid participating to the binding of RAB8A. The centrosomal retention of the CP110 cilia formation-suppressor along with the cilia defect, yet minor, we observed that controls on the AON treatment are consistent with this view.

Intriguingly, while the majority of fibroblasts from individuals homozygous for the c.4723A > T mutation were able to produce a primary cilium upon serum starvation, we observed a highly significant elongation of cilia. Axonemal elongation with normal or abnormal ultrastructure has been originally reported in kidney tissues, cultured kidney-derived cells and skin-derived fibroblasts from individuals or animal models with cystic kidney diseases involving *BBS4* (MIM600374), *NEK8* (MIM609799, NPHP9), *KIF7* (MIM611254, JBTS12), *TMEM67* (MIM609884, MKS3), *KIAA0556* (MIM616650, JBTS26) [38,39,40,41,42,43,44], and in fibroblasts from individuals affected with rod-cone dystrophy and hearing loss due to *CEP78* mutation [45]. Recently, *CEP290*-associated cilia elongation has been described in primary renal epithelial cells from three individuals affected with Joubert syndrome. Two of them were compound heterozygous siblings carrying the c.2817G > T (splice alteration and p.K939N) and c.2848insC (p.Q950Pfs*6) mutations in exon 25 and 26, respectively [46]. The skipping of either exon would preserve the reading frame, leading to the production of a minimally shortened protein that would lack part of the NPHP5 and/or CCD2D2A binding domains. Consistent with positive ciliation, hURECs derived from one of the two siblings expressed minimal amounts of a CEP290 product as determined by western blot analysis. But, the third individual was a sporadic case homozygous for the c.5668G > T (p.G1890*) mutation in exon 41. This in-frame exon has been reported to undergo endogenous basal exon skipping in fibroblasts [20], producing a minimal loss of the microtubule binding domain. Yet, consistent with tissue variability of *CEP290* pre-mRNA splicing [10,21,33,47], RT-PCR analysis of hURECs from the patient harboring elongated cilia showed no skipping of exon 41, Western blot analysis and immunocytochemistry detected no CEP290. Furthermore, siRNA-mediated knockdown of CEP290 led to elongated cilia [46].

To our knowledge, this is the first study showing elongated cilia in fibroblasts for individuals affected with non-syndromic retinal diseases rather than an impairment of cilia formation [8,10,12,21,32]. This observation in two unrelated individuals homozygous for the same mutation strongly supports genotype–phenotype correlation.

It has been proposed that the length of the axoneme which elongates from the basal body is regulated by the balance between the rates of anterograde and retrograde IFT [48]. Whether axonemal elongation in fibroblasts from patients carrying the c.4723A > T mutation is due to alteration of IFT certainly merits consideration. In particular, addressing this question might help in understanding the relation between the abundance of the CEP290^∆36aa^ protein isoform and ciliation and whether AON-mediated axonemal shortening of patient cell cilia may be regarded as a positive read-out of treatment efficiency.

In summary, here we report expression of a PTC-free *CEP290* mRNA resulting from endogenous and selective exclusion of exon 36 encompassing the founder *CEP290* c.4723A > T nonsense mutation in two apparently unrelated individuals. We show that a CEP290 isoform is produced that localizes to centrosomes and that cilia are produced upon serum starvation, yet improperly, as demonstrated by significant axonemal elongation. We demonstrate that increasing the quantity of the *CEP290* mRNA lacking exon 36 through the use of AON increased the abundance of the CEP290 product at the centrosome and allowed axonemal shortening. However, while a moderate quantity of CEP290 product ameliorated cilia formation, a high abundance compromised cilia formation. Whether this would occur in photoreceptor cell is an important question, especially knowing that mRNA metabolism is higher in the retina than in any other cell type [49], that *CEP290* transcripts are far more expressed in neural retina compared to other tissues and organs [19] and that *CEP290* pre-mRNA splicing in iPSC-derived 3D optic cups has been shown to differ significantly from that observed in fibroblasts [10]. AON-mediated skipping of exon 36 in iPSC-derived retinal organoids from individuals carrying the c.4723A > T mutation would certainly merit consideration to address this burning question.

## Figures and Tables

**Table 1:** Impact of the c.4723A > T mutation on exonic splicing silencer (ESS) and exonic splicing enhancer (ESE) motifs within the *CEP290* exon 36.

	Nucleotide	EX-SKIP Predictions			HOT-SKIP Predictions			Skipping Predictions of Mutant Allele Compared to WT Allele
		ESS	ESE	ESS/ESE	ESS	ESE	ESS/ESE	
c.4723 (exon 36)	*A*	12	88	*0.14*	2	17	*0.12*	-
	**T**	19	75	**0.25**	9	4	**2.25**	**Higher chance**
	G	13	87	0.15	3	16	0.19	Higher chance
	C	11	82	0.13	1	11	0.09	Lower chance

Nucleotide change effect at position c.4723 on ESS and ESE motifs according to EX-SKIP and HOT-SKIP prediction programs. The wild-type (WT) and mutant alleles identified in this study are marked in italic and bold, respectively. ESS = Exonic splicing silencer; ESE = Exonic splicing enhancer.

## Supplementary Materials

The following are available online at https://www.mdpi.com/2073-4425/10/5/368/s1, Figure S1: Panel of 199 genes involved in retinal dystrophies, Figure S2: Localization of primers used to amplify the full-length and *CEP290* mRNAs lacking exon 36, Table S1: Sequences and positions of sequencing primers, Table S2: Genetic and clinical features of individuals, Table S3: Sequences and positions of RT-PCR primers, Table S4: Sequences and positions of RT-qPCR primers.
