# Supplementary material for: AON-Mediated Exon Skipping to Bypass Protein Truncation in Retinal Dystrophies Due to the Recurrent CEP290 c.4723A > T Mutation. Fact or Fiction?
Source: Genes (Basel). 2019 May 14;10(5):368. doi: 10.3390/genes10050368 (PMC6562928; doi:10.3390/genes10050368)
Supplement: Supplementary file 1 [file genes-10-00368-s001.pdf]

**Isolated_LCA**	*AIPL1*	*ALMS1*	*C2ORF71*	*C8ORF37*	*CABP4*	*CCT2*	*CEP290*	*CLUAP1*
	CNGA3	*CNGB3*	*CRB1*	*CRX*	*CWC27*	*CYP1A1*	*CYP4V2*	*GUCY2D*
	IFT140	*IMPDH1*	*IQCB1*	*KCNJ13*	*KCNV2*	*KRT12*	*LCA5*	*LRAT*
	MERTK	*MPDZ*	*NMNAT1*	*NR2E3*	*OFD1*	*OTX2*	*PDE6G*	*PRPH2*
	RD3	*RDH12*	*RPE65*	*RPGRIP1*	*SPATA7*	*TULP1*		
**Syndromic_LCA**	*AHI1*	*ALMS1*	*ARL13B*	*C21ORF2*	*C5ORF42*	*CC2D2A*	*CEP104*	*CEP164*
	CEP290	*CEP41*	*CSPP1*	*IFT140*	*INPP5E*	*INVS*	*IQCB1*	*KIF7*
	LCA5	*NPHP1*	*NPHP3*	*NPHP4*	*POC1B*	*RPGRIP1L*	*SDCCAG8*	*TMEM138*
	TMEM216	*TMEM237*	*TTC8*	*TUBB4B*	*VPS13B*	*WDR19*	*ZNF423*	
**Differential_Diagnosis_LCA**	*ATF6*	*CACNA1F*	*CACNA1F*	*CCT2*	*CLN1/PPT1*	*CLN2/TPP1*	*CLN3*	*CLUAP1*
	CNGA3	*CNGB3*	*GNAT1*	*GNAT2*	*GPR179*	*GRM6*	*LRIT3*	*NYX*
	PDE6C	*PDE6H*	*PRPH2*	*SLC24A1*	*SLC38A8*	*TRPM1*		
**Rod_Cone**	*ABCA4*	*ABHD12*	*ADIPOR1*	*AGBL5*	*AHI1*	*ARL2BP*	*ARL6*	*BBS1*
	BBS2	*BBS5*	*BBS12*	*BEST1*	*C2ORF71*	*C8ORF37*	*C21ORF2*	*CA4*
	CACNA1F	*CC2D2A*	*CEP250*	*CEP290*	*CEP78*	*CERKL*	*CHM*	*CLN3*
	CLRN1	*CNGA1*	*CNGB1*	*CRB1*	*CRX*	*CWC27*	*CYP4V2*	*DHDDS*
	DHX38	*EXOSC2*	*EYS*	*FAM161A*	*FLVCR1*	*FSCN2*	*GNAT1*	*GNPTG*
	GPR125	*GUCA1B*	*HGSNAT*	*HK1*	*IDH3A*	*IDH3B*	*IFT43*	*IFT140*
	IFT172	*IMPDH1*	*IMPG2*	*IQCB1*	*ITM2B*	*KIAA1549*	*KIZ*	*KLHL7*
	LAMA1	*LRAT*	*MAK*	*MERTK*	*MFRP*	*MFSD8*	*MPDZ*	*NEK2*
	NEUROD1	*NPHP4*	*NR2E3*	*NRL*	*OFD1*	*OPN1SW*	*OR2W3*	*PCYT1A*
	PDE6A	*PDE6B*	*PDE6G*	*PIK3R4*	*PITPNM3*	*POMGNT1*	*PRCD*	*PROM1*
	PRPF3	*PRPF31*	*PRPF4*	*PRPF6*	*PRPF8*	*PRPH2*	*RBP3*	*RCBTB1*
	RDH11	*RDH12*	*REEP6*	*RGR*	*RHO*	*RIMS1*	*RIMS2*	*RLBP1*
	ROM1	*RP1*	*RP1L1*	*RP2*	*RP9*	*RPE65*	*RPGR*	*RPGRIP1*
	SAG	*SEMA4A*	*SLC7A14*	*SLC24A1*	*SNRNP200*	*SPATA7*	*SRD5A3*	*TOPORS*
	TTC8	*TTLL5*	*TTPA*	*TUB*	*TULP1*	*USH2A*	*WDR19*	*ZNF408*
	ZNF513							
**Cone_Rod**	*ABCA4*	*ABHD12*	*ACBD5*	*ADAMTS18*	*ADAM9*	*AIPL1*	*ALMS1*	*ATF6*
	BBS1	*BBS5*	*C2ORF71*	*C8ORF37*	*C21ORF2*	*CABP4*	*CACNA2D4*	*CDHR1*
	CEP78	*CERKL*	*CNGA3*	*CNGB3*	*CNNM4*	*CRX*	*FAM161A*	*GNAT2*
	GUCA1A	*GUCY2D*	*KCNV2*	*LCA5*	*NMNAT1*	*NR2E3*	*PCYT1A*	*PDE6C*
	PDE6H	*PITPNM3*	*POC1B*	*PROM1*	*PRPH2*	*RAB28*	*RASSF8*	*RAX2*
	RDH5	*RDH12*	*RIMS1*	*RIMS2*	*RPE65*	*RPGR*	*RPGRIP1*	*SEMA4A*
	SPATA7	*SRD5A3*	*TTLL5*	*TULP1*	*UNC119*	*USH2A*		
**Macular_Dystrophy**	*ABCA4*	*ATXN7*	*BEST1*	*C1QTNF5*	*CDH3*	*CNGA3*	*CRB1*	*DRAM2*
	EFEMP1	*ELOVL4*	*FSCN2*	*GUCA1A*	*GUCA1B*	*IMPG1*	*IMPG2*	*MFSD8*
	PROM1	*PRPH2*	*RLBP1*	*RP1L1*	*RPGR*	*TIMP3*		
**Achromatopsia**	*ATF6*	*CACNA1F*	*CLUAP1*	*CNGA3*	*CNGB3*	*GNAT2*	*NDP*	*PDE6C*
	PDE6H							
**Dyschromatopsies**	*OPN1LW*	*OPN1MW*	*OPN1SW*					
**CSNB**	*CABP4*	*CACNA1F*	*GNAT1*	*GNB3*	*GRK1*	*GPR179*	*GRM6*	*HK1*
	LRIT3	*NYX*	*PDE6B*	*RHO*	*SAG*	*SLC24A1*	*TRPM1*	
**RS**	*RS1*	*CRB1*						
**Choroideremia**	*CHM*							
**Others**	*PAX2*	*RBP4*	*GPATCH11*					

Figure S1. Panel of 199 genes involved in retinal dystrophies. LCA: Leber congenital amaurosis; CSNB: congenital stationary night blindness; RS: retinoschisis.

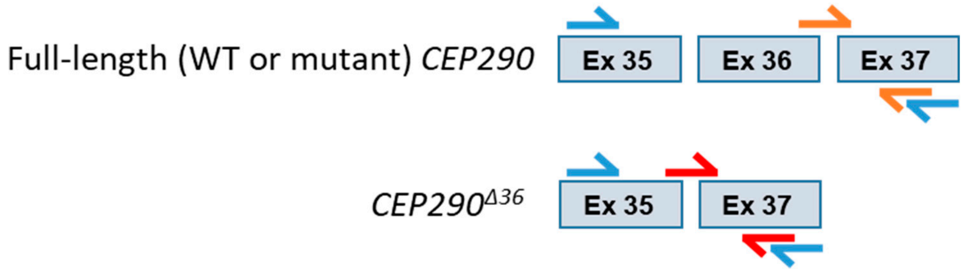

Figure S2. Localization of primers used to amplify the full-length and *CEP290* mRNAs lacking exon 36. In blue, RT-PCR primer pairs used to amplify the full-length and skipped *CEP290* transcripts. In orange, RT-qPCR primer pairs used to amplify the full-length (wild-type, WT or Mutant) *CEP290* transcript. In red, RT-qPCR primer pairs used to amplify the *CEP290* transcript deleted of exon 36 (*CEP290*$^{\Delta 36}$), respectively.

Table S1. Sequences and positions of sequencing primers.

Targeted region	Couple	Position	Sequence (5'>3')
CEP290 Exon 36	*forward*	Intron 35	ACAATTTAAGATATAGTTTCG
	reverse	Intron 36	AACAAAAAGGGTAACTTC

Table S2. Genetic and clinical features of individuals.

Individual	Cell line	Gender	Age* (years)	*CEP290* mutations	Ocular phenotype
Control 1	C1	Female	8	None	No overt pathology
Control 2	C2	Male	10	None	No overt pathology
Control 3	C3	Female	13	None	No overt pathology
Patient 1	P1	Male	34	c.4723A>T (p.Lys1575*)<br>c.4723A>T (p.Lys1575*)	*EOSRD*
Patient 2	P2	Male	24	c.4723A>T (p.Lys1575*)<br>c.4723A>T (p.Lys1575*)	*LCA*

EOSRD: Early onset and severe retinal dystrophy; LCA: Leber congenital amaurosis

*age at which the dermal biopsy and the last clinical examination were performed.

Table S3. Sequences and positions of RT-PCR primers.

Targeted region	Couple	Position	Sequence (5'>3')
CEP290 Exon 36	*forward*	Exon 35	CCACTGCAGAAAGAGAAAAGC
	reverse	Exon 37	TTAGTTTGACCAAGAGTGAGGAA

Table S4. Sequences and positions of RT-qPCR primers. "Full-length" refers to the wild-type or mutant *CEP290* transcript, whereas $CEP290^{\Delta36}$ refers to *CEP290* transcript deleted of exon 36.

Targeted region	Couple	Position	Sequence (5'>3')
« full-length » *CEP290* (exon 36)	*forward*	Jonction exon 36/exon 37	AACAAACGGCTTGGGATTTAATGA
	reverse	Exon 37	TTTGACCAAGAGTGAGGAAAGAGA
$CEP290^{\Delta36}$	*forward*	Jonction exon 35/exon 37	AAAGCCAGAGAGGATTTAATGAAAC
	reverse	Exon 37	TTGACCAAGAGTGAGGAAAGAG
